# Prevalence of hypertension, diabetes mellitus, and their risk factors in an informal settlement in Freetown, Sierra Leone: a cross-sectional study

**DOI:** 10.1186/s12889-024-18158-w

**Published:** 2024-03-13

**Authors:** Ibrahim Franklyn Kamara, Sia Morenike Tengbe, Abdulai Jawo Bah, Innocent Nuwagira, Desta Betula Ali, Fanny F. Koroma, Rugiatu Z. Kamara, Sulaiman Lakoh, Santigie Sesay, James B. W. Russell, Sally Theobald, Mary Lyons

**Affiliations:** 1 World Health Organization Sierra Leone, 21A-B Riverside Drive, Off Kingharman Road, Freetown, Sierra Leone; 2 Ministry of Health, 4th Floor, Youyi Building, Freetown, Sierra Leone; 3 College of Medicine and Allied Health Sciences, University of Sierra Leone, A.J.Momoh Street, Freetown, Sierra Leone; 4 United States CDC Country Office, EOC, Wilkinson Road, Freetown, Sierra Leone; 5 Liverpool School of Tropical Medicine, Pembroke Place, Liverpool, UK

**Keywords:** Hypertension, Diabetes, Prevalence, NCD risk factors, Informal Settlement, Sierra Leone

## Abstract

**Background:**

Noncommunicable diseases (NCDs), especially hypertension and diabetes mellitus are on the increase in sub-Saharan Africa (SSA). Informal settlement dwellers exhibit a high prevalence of behavioural risk factors and are highly vulnerable to hypertension and diabetes. However, no study has assessed the prevalence of hypertension, diabetes, and NCDrisk factors among informal settlement dwellers in Sierra Leone. We conducted a study in June 2019 to determine the prevalence of hypertension, diabetes, and NCD risk factors among adults living in the largest Sierra Leonean informal settlement (KrooBay).

**Methods and materials:**

We conducted a community-based cross-sectional survey among adults aged ≥ 35 years in the KrooBay community. Trained healthcare workers collected data on socio-demographic characteristics and self-reported health behaviours using the World Health Organization STEPwise surveillance questionnaire for chronic disease risk factors. Anthropometric, blood glucose, and blood pressure measurements were performed following standard procedures. Logistics regression was used for analysis and adjusted odd ratios with 95% confidence intervals were calculated to identify risk factors associated with hypertension.

**Results:**

Of the 418 participants, 242 (57%) were females and those below the age of 45 years accounted for over half (55.3%) of the participants. The prevalence of smoking was 18.2%, alcohol consumption was 18.8%, overweight was 28.2%, obesity was 17.9%, physical inactivity was 81.5%, and inadequate consumption of fruits and vegetables was 99%. The overall prevalence of hypertension was 45.7% (95% CI 41.0-50.5%), systolic hypertension was 34.2% (95% CI 29.6–38.8%), diastolic blood pressure was 39.9% (95% CI 35.2–44.6), and participants with diabetes were 2.2% (95% CI 0.7–3.6%). Being aged ≥ 55 years (AOR = 7.35, 95% CI 1.49–36.39) and > 60 years (AOR 8.05; 95% CI 2.22–29.12), separated (AOR = 1.34; 95% 1.02–7.00), cohabitating (AOR = 6.68; 95% CL1.03-14.35), vocational (AOR = 3.65; 95% CI 1.81–7.39 ) and having a university education (AOR = 4.62; 95% CI 3.09–6.91) were found to be independently associated with hypertension.

**Conclusion:**

The prevalence of hypertension,and NCD risk factors was high among the residents of the Kroobay informal settlement. We also noted a low prevalence of diabetes. There is an urgent need for the implementation of health education, promotion, and screening initiatives to reduce health risks so that these conditions will not overwhelm health services.

**Supplementary Information:**

The online version contains supplementary material available at 10.1186/s12889-024-18158-w.

## Introduction

The world is facing an increasing burden of non-communicable diseases (NCDs) associated with changing diets and lifestyles linked to economic development, epidemiological transition, and demographic variations [1–3]. NCDs are a global public health concern as they are responsible for 41 million annual deaths, equivalent to 74% of all deaths worldwide. Each year, 17 million people die from an NCD before the age of 70, and 86% of these premature deaths occur in low- and middle-income countries. Annual deaths from NCDs are projected to escalate to 52 million by 2030 [4, 5]. More alarming is that 80% of all NCD deaths occur in low- and middle-income countries where more than 70% of the world’s poorest live, many in informal settlements [6–8].

The main drivers for increasing rates of hypertension and diabetes in informal settlement populations are urbanization, globalization, and lifestyle factors such as smoking, harmful use of alcohol, low intake of fruits and vegetables, and physical inactivity [9]. Furthermore, informal settlement dwellers often face economic precarity, challenging living conditions, and lack of servicesfacilities, and availability of fresh local produce making them highly vulnerable to developing hypertension and diabetes [10].

Hypertension (high blood pressure) is a chronic medical condition and a primary risk factor for the world’s biggest killer - cardiovascular disease [11]. More than 1 billion of the world’s population had hypertension in 2015, with the highest prevalence in sub-Saharan Africa (SSA) [12–14]. The prevalence of hypertension is increasing in formal urban settings. A recent study conducted among perceived healthy Ghanaian adults documented a high prevalence of unrecognized hypertension [15]. Hypertension used to be considered a condition linked to affluence, but over the last two decades, there has been a paradigm shift with a higher prevalence now seen in people of low socioeconomic status and deprived sections of society, especially those living in informal settlements [16–18]. Studies in Burkina Faso, Tanzania, Uganda, Kenya, Nigeria, and Ghana have all shown a relatively high prevalence of hypertension among people in informal settlements [19, 20]. Additionally, informal settlement dwellers also experience a high prevalence of diabetes [21].

Type 2 diabetes mellitus is a metabolic disease characterized by high blood glucose that results from a decrease in insulin or tissue resistance to insulin [22]. Globally, more than 540 million people live with diabetes with 14 million of these live in SSA [23, 24], and this is expected to rise to 34.2 million by 2040 [14]. In 2021 alone, 6.7 million global deaths were due to diabetes [24]. The World Health Organization has postulated that diabetes will be the seventh leading cause of death by 2030 and by 2045, 626.6 million people will be affected worldwide [24, 25]. A recent study conducted in urban settlements in South Africa showed a high prevalence of pre-diabetes (67%) and diabetes (22%) [26]. Diabetes not only affects the wealthy but also the urban poor, and low socioeconomic status has been associated with the development of diabetes [27]. There appears to be a higher prevalence of diabetes in populations living in informal settlements within cities than in more formal areas in SSA countries such as Kenya, Ghana, and Nigeria [28–30].

In Sierra Leone, data about the burden of hypertension and diabetes is mainly obtained from facility-based studies [31]. However, there is a lack of accurate data about the prevalence of hypertension, diabetes, and NCDs risk factors in the country. A recent study by Russell and colleagues, using a community sample that was not population-based, showed a high prevalence of hypertension (35.3%) and diabetes (8.3%) among adults as part of a screening and awareness programme for NCDs in the capital city of Freetown [32]. No study has provided an estimate of the burden of hypertension, diabetes, and NCD risk factors in informal settlements. We conducted a population-based study in June 2019 to determine the prevalence of hypertension, diabetes, and NCD risk factors among adults living in the largest informal settlement in Freetown, Sierra Leone. We also check for any association between hypertension and NCD risk factors. The findings from our study will be invaluable for planning health service requirements for these populations.

## Methods

### Study design, setting, and population

This was a cross-sectional population-based analytic study. Sierra Leone is a coastal West African country sharing borders with Liberia and Guinea [33]. The majority of the country’s morbidity and mortality is attributed to communicable diseases [34]. The country is urbanizing at a rate of 3% every year, and many residents in the capital city Freetown live in informal settlements [35].

**Fig. 1:**
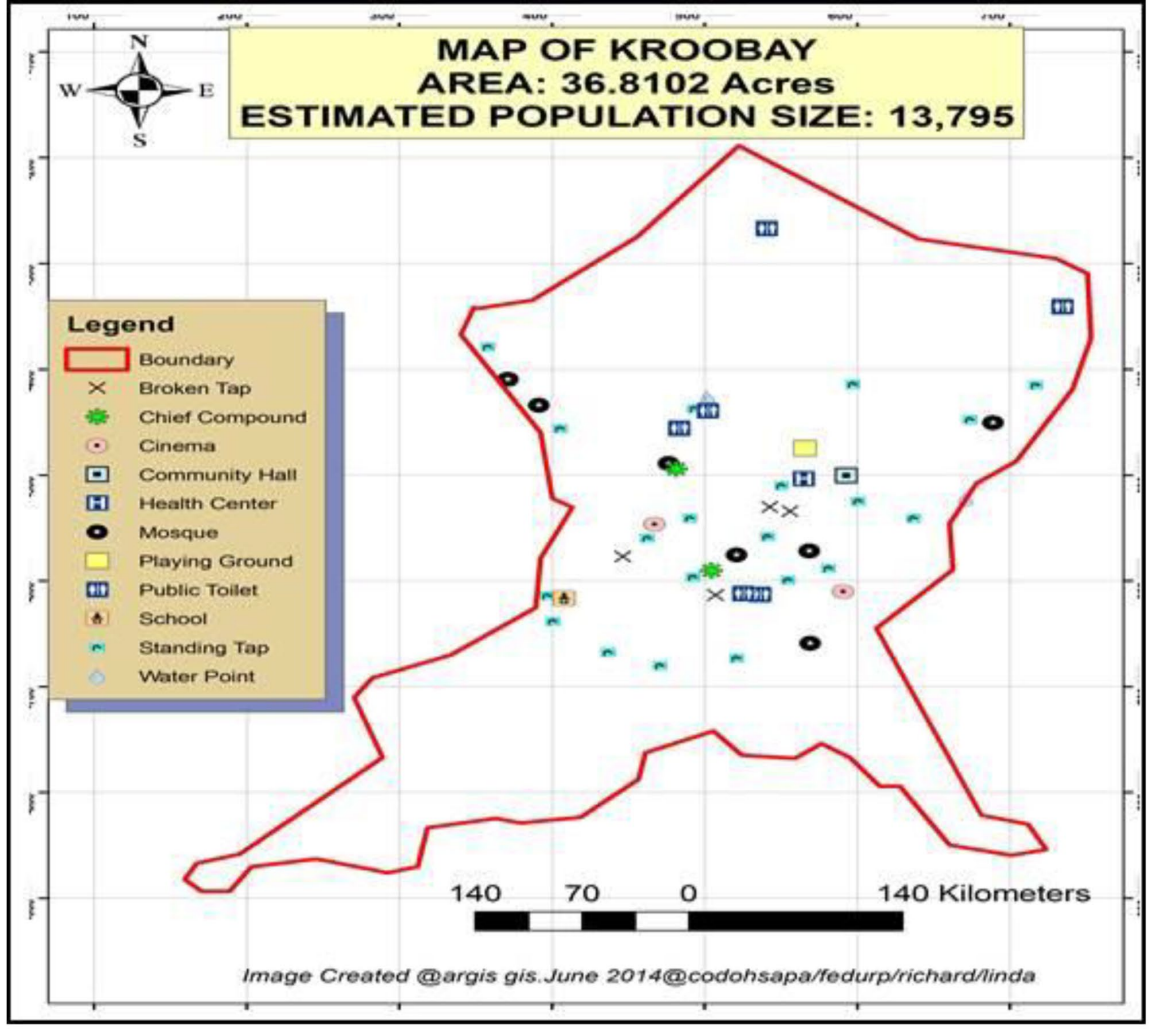
Map showing different strategic points in KrooBay informal settlement, Freetown, Sierra Leone, 2019

This study was conducted in the KrooBay community, which is the largest informal settlement in Freetown (Fig. 1). It is one of the 27 informal settlements that are recognized by the Freetown City Council at the time of the study [36]. It has a land space of 30 acres, an unplanned road network, and is prone to disasters such as fire and flooding. We included adults who had lived in the KrooBay community for at least a year and were aged ≥ 35 years. This age was selected because hypertension and diabetes are rare in those aged under 35 [37].

### Sample size calculation and sampling

The formula (N = Z^2^ x (P)x (1-P)/ d^2^) was used to calculate the estimated sample size [38]. The sample size was calculated with the estimated prevalence of hypertension (35%) in Sierra Leone (P) [39], a precision of 5% (d), and a 95% confidence limit. The minimum estimated sample size calculated was 418 participants which was inclusive of an added 10% for missing and incomplete data.

At the time of the study, KrooBay was randomly selected from the 27 informal settlements in Freetown by simple random balloting from a non-transparent bag containing papers with the names of all 27 informal settlements. The community was divided into five geographic strata using previously established zones.

At least 83 eligible participants were sampled from each of the five zones to achieve the estimated sample size. The first sampling house in each zone was close to a prominent point (for example school, church, or health centre), after which the team moved two consecutive houses to the right and continued in this manner until the sample size for that zone was achieved. This approach minimizes the risk of selection bias and works well in an unplanned community.

### Data collection

We included adults aged ≥ 35 years who were permanent residents in KrooBay who were well and gave their consent to participate. We excluded pregnant women or lactating mothers, the bedridden and those severely ill to prevent discomfort and anxiety, and those who fell below the age of 35 years. To ensure adequate representation from the community, a maximum of three eligible people were invited to participate in the study from each selected household.

Data collection was undertaken between 1st June to 30th June 2019 and was undertaken by medical doctors (2) and nurses (3) with expertise in the measurement of blood pressure, blood glucose levels, and anthropometric parameters. An adapted World Health Organization (WHO) STEPwise questionnaire was used to collect data and was pretested before use (Annexe 1).

### Completion of the adapted WHO STEPwise surveillance questionnaire for chronic disease risk factors

A face-to-face interview lasting between 30 and 45 min was conducted in each participant’s house. The adapted WHO STEPwise surveillance questionnaire for chronic disease risk factors [40] was used to collect data on smoking, alcohol consumption, fruit and vegetable consumption, and physical activity. Measurements of blood pressure, random blood glucose, height, and weight were taken from each participant. All questionnaires were checked for completeness, consistency, and accuracy.

### Measurements and definitions

The instruments used to measure blood pressure, blood glucose, weight, and height were all validated and staff trained in their use.

Height was measured to the nearest 0.1 cm using a portable Seca stadiometer. Weight was measured to the nearest 0.1 kg using a calibrated Seca weight scale with the participant dressed in light clothing and without shoes.

Blood pressure was measured on the non-dominant arm using an automatic OMRON M6 digital monitor [41]. Two separate readings were recorded with the subjects sitting, and at least five minutes apart, and the average was considered as the participant’s final blood pressure. Hypertension was defined as Systolic blood pressure (SBP) ≥ 140 mmHg and/or diastolic blood pressure (DBP) ≥ 90 mmHg or being on anti-hypertensive medications (Table 1). Blood pressure was classified according to the Seventh Joint National Committee on Prevention, Detection, Evaluation, and Treatment of High Blood Pressure (JNC VII) into normal (systolic BP < 120 and diastolic BP < 80), pre-hypertensive (SBP 120–139 or DBP 80–89), stage 1 (SBP 140–159 or DBP 90–99) and stage 2 (SBP ≥ 160 or DBP ≥ 100) hypertension [42].

We measured random blood glucose using an eBSensor eB-G glucometer. A minimal amount of capillary blood was collected on the fitted strip in the glucometer. Diabetes was defined as a single high random blood glucose ≥ 11.1mmol/L (200 mg/dl) or being on medications for diabetes (Table 1).

Tobacco use- and alcohol consumption were self-reported by the respondent. An unhealthy diet was defined as the consumption of fewer than five portions of fruits and/or vegetables in a day for at least five days in an average week and insufficient physical activity is less than 75 min of vigorous-intensity or 150 min of moderate-intensity physical activity per week.

**Table 1:** Operational definitions

Terms	Meaning
Hypertension	Systolic blood pressure ≥ 140 mmHg and/or diastolic blood pressure ≥ 90 mmHg or being on anti-hypertensive medications [13]
Diabetes	Single random blood glucose ≥ 11.1mmol/L (200 mg/dl) or being on medications for diabetes mellitus [13]
Overweight	BMI = 25.0-29.9 kg/m^2^ [13]
Obesity	BMI ≥ 30 kg/m^2^ [13]
Tobacco use	Current (smoking) tobacco use as self-reported by the respondent [43]
Alcohol consumption	Current alcohol consumption as self-reported by the respondent
Unhealthy Diet	Consumption of fewer than five portions of fruits and/or vegetables in a day for at least five days in an average week [43]
Insufficient physical inactivity	<75 min of vigorous-intensity or < 150 min of moderate-intensity physical activity per week or its equivalent of the combination [43]

The body mass index (BMI) was defined as a person’s weight in kilograms divided by their height in meters square and categorized accordingly into underweight (< 18 kg/m^2^), normal weight (18–25 kg/m^2^), overweight (25–30 kg/m^2^) and obesity (≥30 kg/m^2^)

### Data analysis

Data statistical analysis was carried out using the IBM Statistical Package for Social Science (SPSS) version 24. All variables were checked to identify outliers, extreme values, and missing data. We cross-checked the source documents to address outliers, extreme values, and missing data, and further queries were corrected using the imputation method.

Multivariable logistic regression analyses were performed by adjusting for all other variables (i.e., age, gender, educational level attained, marital status, smoking, alcohol use, physical inactivity, unhealthy diet, weight, height, and diabetes) excluding the main variable under each investigation. These variables were selected as they are potential risk factors for hypertension based on available evidence.

Before modeling, multicollinearity was checked using the variance inflation factor. All the variables included in the model had VIF less than 10. Multivariable logistic regression was used and adjusted odds ratios (AOR) with 95% confidence intervals (CI) were reported as the measure of association.

### Ethics

The study obtained ethical approval from the Liverpool School of Tropical Medicine and the Sierra Leone Ethics and Scientific Review Committee. All study participants were informed about the study aims including potential benefits and risks associated with participation. Verbal consent to approach participants was sought from the head of each household and eligible participants gave informed written consent before participation. Participants who had hypertension and high blood glucose were referred to the nearby tertiary hospital for further evaluation and management.

## Results

Of the 418 participants, 242 (57.9%) were females. The mean (SD) age of the participants was 44.8 (± 9.7) years. Other details are shown in Table 2.

**Table 2:** Socio-demographic characteristics among adults in the KrooBay community, Freetown, 2019

Variables (*N* = 418)	n	(%)
**Gender**		
Male	176	42.1
Female	242	57.9
**Age group (in years)**		
35–39	170	40.7
40–44	61	14.6
45–49	62	14.8
50–54	35	8.4
55–59	38	9.1
≥ 60	52	12.4
**Religion**		
Muslim	354	84.7
Christian	64	15.5
**Marital status**		
Married/Cohabitating	280	68
Single/separated/divorced/widowed	138	32
**Ethnic group**		
Temne	240	57.4
Limba	42	10.0
Fullah	41	9.8.
Loko	14	3.3
Mende	13	3.1
Creole	6	1.4
Others	62	14.8
**Level of education**		
No formal education	233	55.7
Primary	42	10.0
Secondary/Vocational/ College/University	143	34.3
**Employment status**		
Petty trader/Businessperson/Self-employed	230	55.0
Informal employment	105	25.1
Unemployed	46	11.0
Housewife	11	2.6
Government	10	2.4
Non-government	7	1.7
Retired	7	1.7
Student	2	0.5

**Fig. 2:**
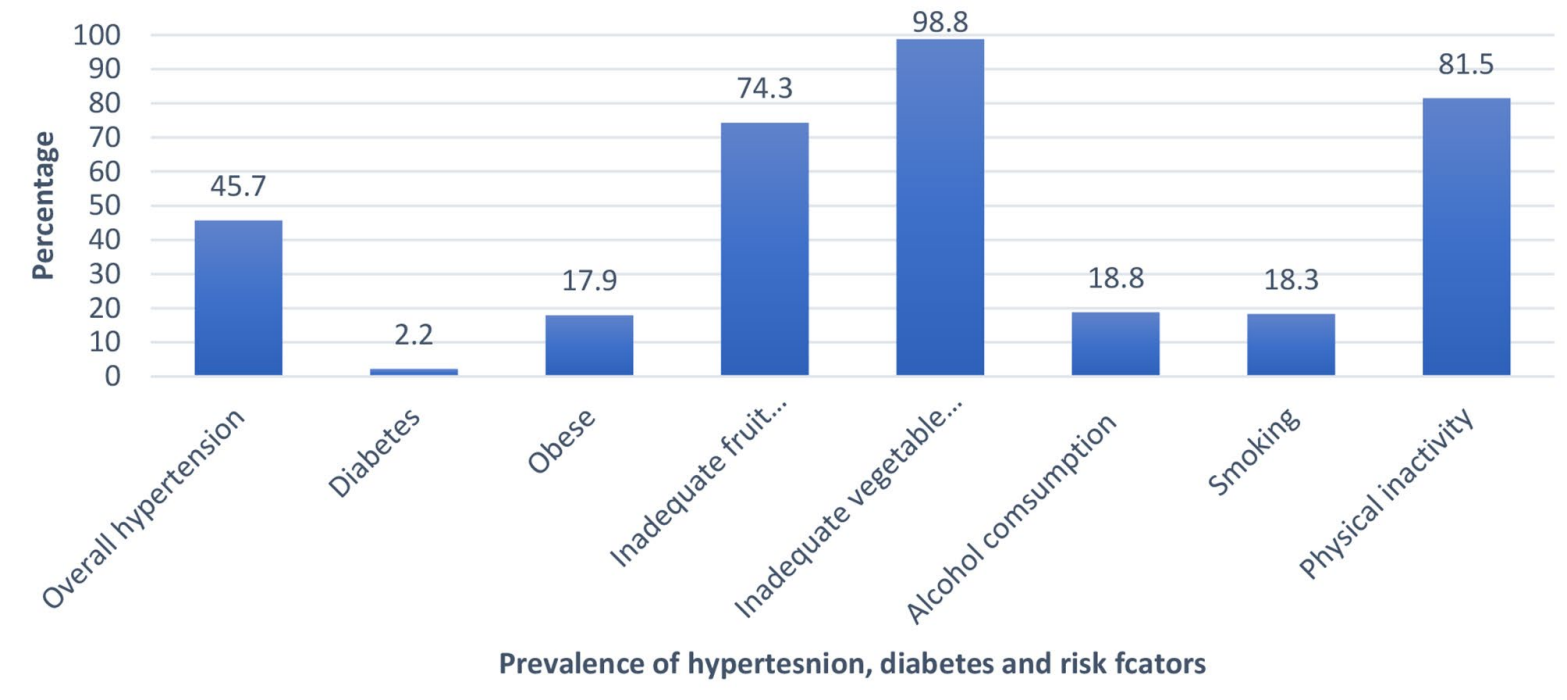
Prevalence of hypertension, diabetes, and their risk factors among adults in the KrooBay community, 2019

The prevalence of hypertension,,and NCD risk factors were high in the KrooBay community. The prevalence of NCD risk factors varied from smoking (18%) to inadequate consumption of vegetables (98.8%). The prevalence of smoking and alcohol consumption was relatively low (Fig. 2).

### Prevalence of NCDs risk factors

**Fig. 3:**
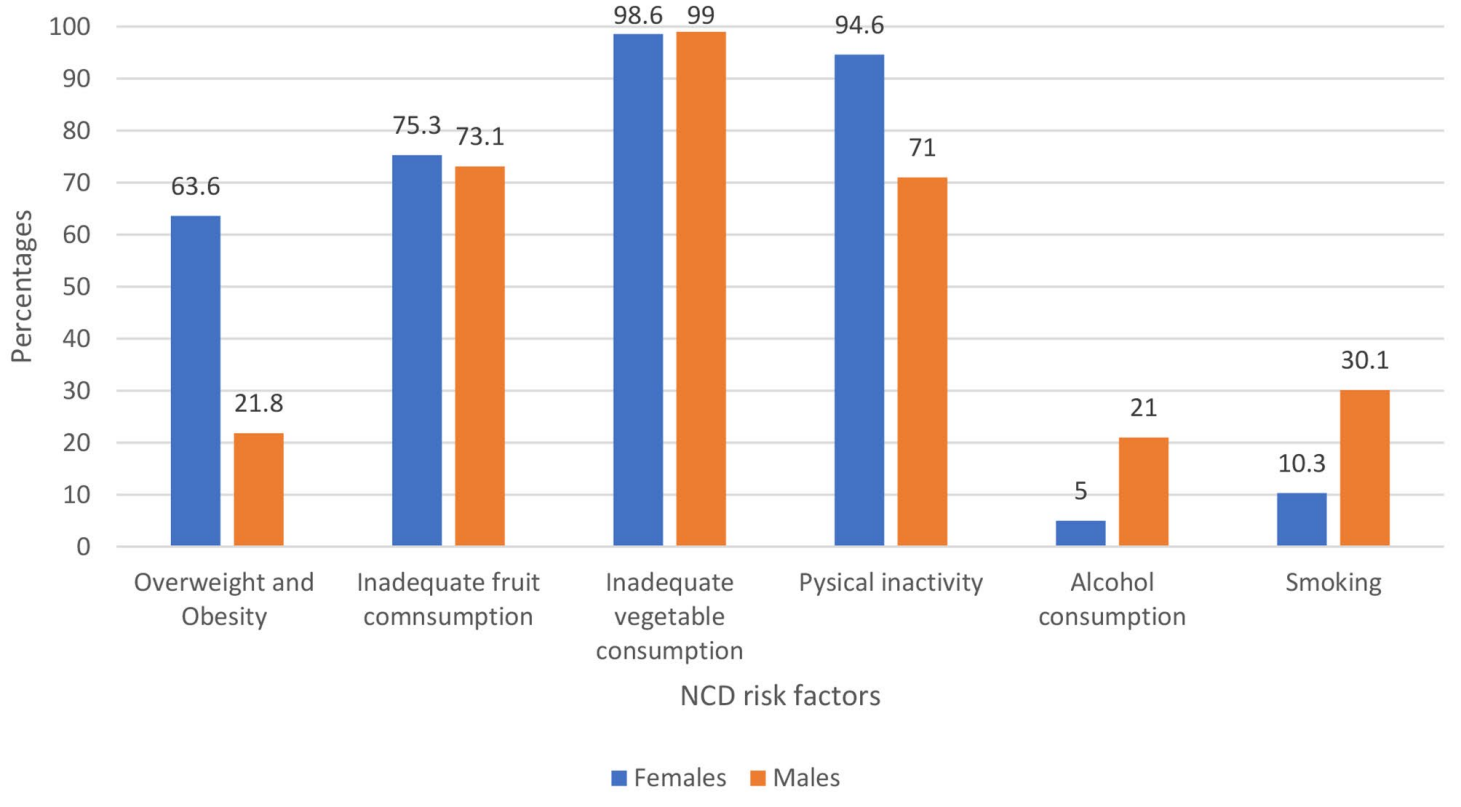
Prevalence of NCDs risk factors by gender among adults in the KrooBay community, 2019

Among the 418 participants in the study, the mean BMI (SD) was 25.7 (5.7) kg/m². Overweight and obesity were prevalent in 46.0% of participants (95% CI 41.1–50.8) (F,. Daily fruit consumption was reported by 74.3% (95% CI 69.1–79.4), However, 75.6% of participants only had one portion of fruit per day, and less than 1% met the recommended five portions. Vegetable consumption was high (98.8%, 95% CI 97.4–100), but 93.1% only consumed one portion daily. Physical inactivity was prevalent in 81.5% (95% CI 81.1–81.8) of adult residents. Alcohol consumption was noted in 18.8% (95% CI 18.5–19.1). Smoking prevalence was 18.2% (95% CI 17.8–18.5) with 81% of current smokers smoking over five cigarettes per day. The prevalence of overweight and obesity, inadequate fruit consumption, and physical inactivity was higher in females than in males ( Fig. 3).

### Prevalence of hypertension

The overall prevalence of hypertension among adults in the KrooBay community was 45.7% (95% CI 41.0-50.5). This prevalence was higher in females (49.6%) as compared to males. Participants aged ≥ 60 years had the highest prevalence (75%). Over a quarter (29.7%) of the participants were prehypertensive, some (21.8%) had stage 1 hypertension and about a quarter (23.9%) had stage 2 hypertension (Table 3). Of those who have hypertension, only a small fraction (14.1%) were on antihypertensive medications. The mean (SD) systolic blood pressure (SBP) was 135 (26.3) mmHg. The prevalence of elevated SBP ≥ 140 mmHg was 34.2% (95% CI 29.6–38.8%). Participants aged ≥ 60 years had the highest prevalence (63.5%) of SBP. elevated SBP was more prevalent among females at 35% (95% CI 31.1–40.3) than males at 32.2% (95% CI 27.6–36.8). Of the 418 participants, 79 (18.9%) were suffering from stage one hypertension and 63 (15.3%) had stage two hypertension as detailed in Table 3. The mean (SD) diastolic blood pressure (DBP) was 87.8% (14.8) mmHg. The prevalence of elevated DBP ≥ 90 mmHg was 39.9% (95% CI 35.2–44.6). The prevalence was higher among females at 44.6% (95% CI 41.6–47.6) than males at 33.5% (95% CI 30.5–36.5). Of the 418 participants, 83 (19.9%) were suffering from stage 1 hypertension and 82 (19.6%) had stage 2 hypertension (Table 3). Participants aged ≥ 60 years accounted for the highest prevalence (67.3%) of elevated DBP.

**Table 3:** Elevated SBP and/or elevated DBP according to the Seventh Joint National Council

	SBP and/or DBP	N(%)	SBP	N (%)	DBP	N (%)
Normal	< 120/<80	103 (24.7)	<120	141 (33.7%)	<80	150 (35.9%)
Pre-hypertension	120–139/80–89	124 (29.7)	120–139	135 (32.3%)	80–89	103 (24.6%)
Stage 1	140–159/90–99	**91 (21.8)**	140–159	**79 (18.9%)**	90–99	**83 (19.9%)**
Stage 2	≥ 160/≥100	**100 (23.9)**	≥160	**63 (15.3%)**	≥ 100	**82 (19.6%)**

### Factors associated with hypertension

The factors significantly associated with hypertension were age, marital status, and level of education as detailed in Table 4. Elderly > 55 years (AOR = 7.35, 95% CI 1.49–36.39) and > 60 years (AOR = 8.05; 95% CI 2.22–29.12), both men and women separated from their marriages (AOR = 1.34; 95% 1.02–7.00) cohabitating (AOR = 6.68; 95% CL1.03-14.35), vocational education (AOR = 3.65; 95% CI 1.81–7.39 ) and university education (AOR = 4.62; 95% CI 3.09–6.91) all showed a significantly higher risk of hypertension.

**Table 4:** Factors associated with hypertension

Variables	Hypertension	Adjusted OR (AOR)	95% CI	*p*-value
Gender	Female	0.96	(0.31–3.04)	0.95
	Male	Reference group		
Age group	≥ 60	8.05	(2.22–29.12)	**< 0.001**
	55–59	7.35	(1.49–36.39)	**0.01**
	50–54	2.51	(0.53–11.82)	0.24
	45–49	4.54	(1.23–16.71)	**0.02**
	40–44	2.83	(0.83–9.63)	0.10
	35–39	Reference group		
Marital Status	Cohabitating	6.68	(1.03–14.35)	**< 0.001**
	Widowed	1.27	(0.27–5.95)	0.76
	Separated	1.34	(1.02–7.00)	**< 0.001**
	Married	0.73	(0.23–2.33)	0.59
	Single	Reference group		
Level of Education	University	4.62	(3.09–6.91 )	**< 0.001**
	Vocational	3.65	(1.81–7.39 )	**< 0.001**
	Secondary	0.88	(0.32–2.42)	0.81
	Primary	1.08	(0.30–3.80)	0.92
	No Education	Reference group		
Recommended vegetables consumption	No	0.86	(0.12–6.39)	0.88
	Yes	Reference group		
Recommended fruits consumption	No	1.33	(0.56–3.15)	0.51
	Yes	Reference group		
Smoking	No	0.72	(0.24–2.22)	0.57
	Yes	Reference group		
Alcohol consumption	No	0.62	(0.14–2.80)	0.54
	Yes	Reference group		
Vigorous exercise	No	0.71	(0.17–2.92)	0.63
	Yes	Reference group		
Obesity	No	2.13	(0.84–5.41)	0.11
	Yes	Reference group		
High Blood Glucose	No	1.40	(0.19–10.53)	0.74
	Yes	Reference group		

Obesity-Body mass index ≥ 30 kg/m2; High blood sugar-random blood sugar ≥ 11.1mmol/ (200 mg/dl); vigorous exercise- 150 min of moderate-intensity activity per week or 75 min of vigorous-intensity activity per week; smoking- Current (smoking) tobacco use as self-reported; Current alcohol consumption as self-reported; recommended vegetables and fruits- five portions per day

### Prevalence of diabetes mellitus

The mean (SD) random blood glucose was 6.47(2.0) mmol/l. The prevalence of random blood glucose ≥ 11.1 mmol/l (suggestive of diabetes) was 2.2% (95% CI 0.7–3.6) mmol/l(Table 5). Diabetes was more prevalent in women at 3.3% (95% CI 2.3–4.4) than in men at 0.6% (95% CI 0.3–1.6). Participants aged ≥ 50 years accounted for most of the cases and the lowest prevalence was seen in participants aged 35–39 years. Of those who have diabetes, only a small fraction (12.5%) were on antidiabetic medications.

**Table 5:** Overall and gender-related prevalence of diabetes among participants in KrooBay, 2019

Overall	2.2 (CI 0.7 -3 0.6)
**Sex**	
Male Female	0.6 (CI 0.3–1.6) **3.3 (CI 2.3–4.3)**

## Discussion

This was the first community-based prevalence study conducted in Sierra Leone that assessed the prevalence of hypertension, diabetes, and NCD risk factors in an informal settlement. Our study found that there was a high prevalence of hypertension, and NCDs risk factors, and a low prevalence of diabetes among adults living in the largest informal settlement (KrooBay) in Sierra Leone.

The prevalence of hypertension among residents in KrooBay was 45.7%. This was higher to the prevalence (34.8%) seen in Sierra Leone’s STEPS survey conducted in 2009, and higher than other studies conducted in the country and Kenya [27, 43, 44, 45]. A study conducted in Ajegunle one of Nigeria’s largest informal settlements documented a lower prevalence (38.2%) of hypertension compared to what we found in KrooBay [16]. This high prevalence of hypertension puts the people in the krooBay community at higher risk of suffering cardiovascular diseases like heart attacks, heart failure, and strokes. In our study, the prevalence of hypertension was higher in females than in males, which was unexpected as the disease tends to be more prevalent in African males as documented in several studies including Kenya [18]. In our study, the increased risk for women might be linked to being overweight and obese, shaped by limited opportunities to exercise, and challenges in being able to access a range of healthy foods. This is in keeping with other studies that have documented increased weight as an independent risk factor for hypertension [46]. Since the prevalence of hypertension is higher in women than in men, innovative strategies for women that support weight loss and access to healthy foods should be prioritised. These should be co-designed with women from Kroo Bay and similar settlements to ensure they meet their needs within the contexts in which they live. Engaging with women leaders and key stakeholders in this process will support the implementation and sustainability of these approaches.

We also found it interesting and rather surprising that the risk of hypertension was higher among the more educated sections of the KrooBay population. We postulated this may be because those with a higher level of education work in more stressful environments. The high workload in these settings might lead to increased psychosocial stress. There is evidence that psychosocial stress is a risk factor associated with hypertension as it affects the nitric oxide system which has a complex mechanism [39]. Furthermore, it is also possible that they also earn more and can afford junk food which can in turn increase their body weight and their risk of suffering from hypertension and other NCDs. This requires further investigation.

There was also a weak association between age, marital status, level of education, and hypertension in our study. We observed that increase in age increases the risk of hypertension. A larger sample size may show a strong association between increased age and increasing risk for hypertension. Several studies conducted in Africa have shown a strong association between hypertension and increase in age [47]. We observed that there were more participants with pre-hypertension (29.7%), followed by stage 2 hypertension (23.9%) and then stage 1 hypertension (21.8%).

The prevalence of diabetes was 2.2%. This is lower than the global prevalence of 6.1% [48]. In our study, females were affected more than males, and this might also be linked to the high prevalence of overweight and obesity among females in the KrooBay community. Being overweight and obese are known to be significant predictors of diabetes [21]. Similar findings were seen in a study conducted in the Kibera informal settlement (Kenya) where a diabetes prevalence of 3.2% was noted [27]. Diabetes prevalence of 11.7% was seen in Agu-Abor and Ugbodogwu informal settlements in Nigeria [30].

Diabetes in deprived and informal settlement communities has been linked to physical inactivity, unhealthy diet, being overweight and obese, and cigarette smoking [10]; and shaped by individual factors, contextual factors, and health system-related factors [49]. We found a relatively high prevalence of the different NCD risk factors, but a lower prevalence of diabetes compared to other countries in West Africa [29]. However, the low prevalence of diabetes in our study might be attributed to the use of random blood glucose measurements. The prevalence may well have been higher if we had recorded fasting blood glucose or glycosylated haemoglobin which are the gold standards used for diagnosing diabetes. Since the prevalence of hypertension, overweight and obesity are high in KrooBay, the prevalence of diabetes will likely increase over time as it has a strong association with hypertension and obesity [10]. In line with the interpretation of our findings, a recent study conducted in India documented a strong association between older age, overweight and obesity, and raised blood pressure as an increased risk for the development of diabetes [50]. It is therefore critical for diabetes prevalence, awareness, screening, and treatment programmes should be prioritised and implemented in Kroobay and other informal settlements. The intervention should be gender aware, and strengthen opportunities for awareness raising and screening. For example, women from the KrooBay community who contact a healthcare facility could be offered screening for diabetes. However, it is worth noting that implementing comprehensive diabetic programmes is challenging in low-income countries like Sierra Leone [51].

The prevalence of NCD risk factors in KrooBay were similar to those found in a peri-urban informal settlement in Lima, Peru [52]. The prevalence of obesity and overweight in KrooBay was 46.6%. This is higher than the national prevalence of 30.2% [53] and also higher than the prevalence (43.5%) documented in a similar study conducted in an informal settlement in Kenya [45]. The high prevalence of obesity and overweight in KrooBay is not surprising, given this is more common in urban areas as compared to rural settings [54], and the challenges associated with daily life within informal settlements contexts including lack of access to fruit and vegetables. The prevalence of alcohol consumption and smoking are 18.8% and 18.2% respectively, and were higher amongst men, and the reasons for this need further exploration.These are similar to the findings in the national STEPS survey where Alcohol consumption was 17.2% and smoking was 22.5% [53]. However, the prevalence obtained in our study is higher as compared to the study done in the Kibera informal settlement where alcohol consumption and smoking were 13.1% and 8.5% respectively [45]. Due to the high prevalence of these risk factors in KrooBay, this vulnerable population is at increased risk of suffering from hypertension and diabetes. There is a need to co-develop gender-aware and context-embedded strategies that operate at the individual, contextual, and health systems levels to reduce risk factors including obesity/overweight and tobacco and alcohol consumption. These include health education and promotion activities on the harmful effects of smoking and alcohol in men’s communal areas like football playgrounds and cinemas.

A nationwide stepwise survey would provide us with a robust countrywide prevalence estimate of hypertension, diabetes, and NCD risk factors that can be used to inform health education and promotion initiatives as well as other NCD-related strategies, policies, and services intervention. Therefore, the STEPS survey must be conducted soonest as it is long overdue and the recommendation is that it should be conducted every 3–5 years.

For the effective implementation of NCDs and their risk factor reduction strategy, we recommend context-specific information, education, and communication materials to be developed and used in the KrooBay community to encourage lifestyle modification within the realities of life in informal settlements, and in turn, reduce the burden of hypertension and diabetes in the community. This should include population-based intervention like the taxation of tobacco and alcohol as stated in the National Tobacco and Nicotine Control Act and alcohol policy. These interventions are effective as stated in the WHO HEART technical package for the control of behavioural risk factors [55]. Additionally, the inclusion of hypertension and diabetes awareness, prevention, detection, and treatment at the primary healthcare level will facilitate these conditions being identified early and managed appropriately. This is effective as stated in the first-ever WHO global report on hypertension [56].

### Strengths and limitations

Our study has several strengths. First, The STEPS methodology is adaptable and flexible enough to be implemented in resource-constrained settings like Sierra Leone. Furthermore, It provides robust data on NCD risk factors which supports countries to track progress against national targets and as such guides the development of policies, strategies, and action plans to reduce NCD morbidity and mortality [57]. Second, we used a representative sampling method to determine the prevalence of hypertension, diabetes, and NCD risk factors and an adapted and validated WHO STEPwise questionnaire for data collection. Third, our study was well-powered as 418 people participated in the study. Fourth, we adhered to the Strengthening the Reporting of Observational Studies in Epidemiology (STROBE) guideline to report our findings [58].

There were a few limitations to our study, first, this is a one-off surveillance approach as the country has to integrate NCD risk-factor surveillance into the national health routine surveillance system. Additionally, the questions and measures used in STEPS and the indicators reported from STEPS surveys need to be periodically reviewed to adapt to the latest scientific standards and policy needs for real-time decision-making [57]. Second, we used a glucometer to measure random blood glucose which cannot make a definitive diagnosis of diabetes. Laboratory investigations such as fasting blood glucose and glycosylated haemoglobin are the gold standard. However, this is an acceptable approach for screening activities and all the participants with a single high RBS were referred to a hospital for confirmation. Furthermore, it has been documented that a single random blood sugar ≥ 5.6 mmol./l is strongly associated with undiagnosed diabetes [59]. Third, we were unable to check for risk factors associated with diabetes as the prevalence was low. This low prevalence might be due to the calculated sample size that was determined by parameters related to hypertension. Fourth, a single contact with participants to make a diagnosis of hypertension is less accurate than two or more contacts in different situations, with blood pressure measured sitting and standing at least 15–30 min apart. However, the method we used is acceptable in screening programmes. It also needs to be noted that our study took place pre-COVID-19, where many risk factors are likely to have been further amplified.

## Conclusions

Our study is the first population-based prevalence study on hypertension, diabetes, and NCD risk factors in an informal settlement in Sierra Leone. We found a high prevalence of hypertension, and NCDs risk factors among adults resident in the KrooBay community. Women appeared to have a greater risk of hypertension and diabetes than men. Furthermore, there were a lot of people in the pre-hypertensive stage which is itself a risk factor for developing hypertension. Hypertension was associated with increasing age, being divorced or separated, and university education.

The prevalence of overweight and obesity, physical inactivity, and inadequate consumption of fruits and vegetables were higher than those found in the 2009 STEPS survey. Alcohol consumption and smoking prevalence were higher compared to other countries in SSA. These are all risk factors for hypertension and diabetes and if contextually appropriate health education and promotion activities are not successfully implemented, hypertension, diabetes and NCD risk factors burden will increase in this community and other similar contexts.

### Electronic supplementary material

Below is the link to the electronic supplementary material.

Supplementary Material 1

## Data Availability

The datasets used and analyzed during the current study are available from the corresponding author upon reasonable request.

## Abbreviations

BMI: Body Mass Index
COPD: Chronic Obstructive Pulmonary Diseases
CVDs: Cardiovascular Diseases
DBP: Diastolic Blood Pressure
DM: Diabetes Mellitus
FBS: Fasting blood sugar
JNC: Joint National Committee
NCDs: Noncommunicable Diseases
NICE: National Institute for Health and Care Excellence
RBS: Random blood sugar
SBP: Systolic Blood Pressure
SSA: Sub-Saharan Africa
STEPS: STEPwise Approach to surveillance for chronic disease risk factors
WHO: World Health Organization
