# Supplementary material for: Prevalence of hypertension, diabetes mellitus, and their risk factors in an informal settlement in Freetown, Sierra Leone: a cross-sectional study
Source: BMC Public Health. 2024 Mar 13;24:783. doi: 10.1186/s12889-024-18158-w (PMC10935859; doi:10.1186/s12889-024-18158-w)
Supplement: Supplementary file 1 — Supplementary Material 1 [file 12889_2024_18158_MOESM1_ESM.docx]

## Supplementary Material 1: World Health Organization adapted STEPS questionnaire

Prevalence of Hypertension, Diabetes Mellitus, and Noncommunicable Diseases Risk Factors among Adults (35-64 years old) in an informal settlement in Freetown, Sierra Leone.

| **Survey Information** | |
| --- | --- |
| **Location and Date** | **Response** |
| Zone Number |  |
| Interviewer ID |  |
| Date of completion of the instrument |  |
|  |  |
| **Consent, Interview Language** | |
| Consent has been read and obtained | Yes  No if No complete consent |
| Interview Language | English  Krio |
|  | |
| **Step 1 Demographic Information** | |
| **Question** | **Response** |
| Sex | Male  Female |
| How old are you? |  |
| Religion | Christian  Muslim  Others, Please specify…………………… |
| What is the highest level of education you have completed? | No Formal Schooling  Primary School  Secondary School  Vocational Institution  College/University  Post Graduate Degree |
| What is your *ethnic/tribal* background? | Fullah  Krio  Limba  Loko.  Mende  Temne  Other, Please Specify…………………….. |
| What is your marital status? | Single  Married  Separated  Divorced  Widowed  Cohabitating |
| Which of the following best describes your main work status? | Government employee  Non-government employee  Self-employed  Trader  Businessman  Student  Housewife  Retired  Unemployed (able to work)  Unemployed (unable to work) |
| Can you give an estimate of the monthly household income? | ≤ Le 500,000  More than Le 500,000 ≤ Le 750,000  More than Le 750,000 ≤ Le 1,000,000  More than Le 1,000,000 ≤ Le 2,000,000  More than Le2.000,000 ≤ Le 3,000,000  More than Le 3,000,000  Don't Know |
| What kind of material is your house made of? | Concrete  Zinc  Wood  Mud blocks  Don’t Know |
| Do you own or rent this House? | Own outright  Part owns and part rents  Rents  Lives here rent free |
| How many cars, motorbikes or Keke are owned or available for use by the members of this household? | Cars  Motorbikes  Keke |
|  | |
| **Behavioral Measurement** | |
| **Tobacco Use** | |
| **Question** | **Response** |
| Do you currently smoke | Yes  No |
| If yes, what do you smoke? Tick all that apply) | Cigarette  Shisha  Marijuana  Cigar  Pipe full of tobacco  Others (please specify) |
| Do you currently smoke tobacco product? | Yes  No, If No skip to the next question |
| On average, how many of the following do you smoke daily/week? | \| Item \| Daily \| Weekly \| \| --- \| --- \| --- \| \| Cigarette \|  \|  \| \| Shisha \|  \|  \| \| Marijuana \|  \|  \| \| Cigar \|  \|  \| \| Pipe full of tobacco \|  \|  \| \| Others (please specify)  …………………. \|  \|  \| |
| Does someone else smoke **in** your home? | Yes  No |
| Do you use wood as a source of fuel for cooking? | Yes  No |
| If yes - where is cooking with wood done in your house | Inside the house  <5 Metres from the house  5 Metres to 10 Metres from the house  >10 Metres from the house |
|  | |
| **Alcohol Consumption** | |
| **Question** | **Response** |
| Have you consumed any alcohol such as beer, Cider, wine, spirits, Local alcohol- "omoleh" or palm wine in the last seven days? | Yes  No |
| If yes - how frequently do you drink | Daily  5-6 days per week  3-4 days per week  1-2 days per week  1-3 days per month  Less than once a month  Occasionally |
| What do you drink? | Beer  Cider  Wine  Spirit  Local alcohol- Omoleh  Palm wine |
| How many do you drink? i.e. Number of pints or glass |  |
| **Diet** | |
| **Question** | **Response** |
| In a typical week, on how many days do you eat fruit? Eg mango, Banana, Apple Oranges and Lemon etc. |  |
| How many servings of fruit do you eat in a typical day? |  |
| In a typical week, on how many days do you eat vegetables? Eg Cassava Leaves, Potato leaves, Cassava, Potatoes, onions and pepper etc. |  |
| How many servings of vegetables do you eat in a typical day? |  |
|  | |
| **Physical Activities** | |
| **Question** | **Response** |
| Do you ever undertake vigorous physical activity for at least ten minutes that makes you sweat and your heart beat faster | Yes  No |
| In a typical week, on how many days do you do vigorous-intensity activity? |  |
| What sort of vigorous activity do you do? (tick all that apply) | Brisk walking  Running  Jogging  Swimming  Riding a Bicycle  Weight lifting  Others, please specify………………….. |
|  | |
| **History Blood Pressure** | |
| **Question** | **Response** |
| Have you ever been told by a doctor or other health worker that you have raised blood pressure or hypertension? | Yes  No |
| If yes, how long have you had high blood pressure? | Months  Years |
| Do you take any drugs (medication) for raised blood pressure prescribed by a doctor or other health worker? | Yes  No |
| Have you ever seen a traditional healer for raised blood pressure or hypertension? | Yes  No |
| Are you currently taking any herbal or traditional remedy for your raised blood pressure? | Yes  No |
|  | |
| **History of diabetes mellitus** | |
| **Question** | **Response** |
| Have you ever been told by a doctor or other health worker that you have raised blood sugar or diabetes? | Yes  No |
| Do you take any drugs (medications) for diabetes prescribed by a doctor or other health worker? | Yes  No |
| Have you ever seen a traditional healer for diabetes or raised blood sugar? | Yes  No |
| Are you currently taking any herbal or traditional remedy for your diabetes? | Yes  No |
|  | |
| **Step 2 Physical Measurements** | |
| **Blood Pressure Readings** | |
| Reading 1 | Systolic  Diastolic. mmHg |
| Reading 2 | Systolic  Diastolic. mmHg |
|  |  |
| For women: Are you pregnant? | Yes  No |
| **Weight and Height** | |
| Height | In Metres |
| Weight | In Kilograms |
|  | |
| **Step 3 Biochemical Measurement** | |
| **Random Blood Sugar** | |
| **Question** | **Response** |
| When did you last have something to eat? | Less than 12 hours  More than 12 hours |
| Blood Glucose Value | Mmol/L |
